# Predictive factors and clinicopathological characteristics of outcome in poorly differentiated thyroid carcinoma: a single-institution study

**DOI:** 10.3389/fonc.2023.1102936

**Published:** 2023-07-07

**Authors:** Zheng Wan, Bing Wang, Jing Yao, Qing Li, Xin Miao, Yanbing Jian, Sisi Huang, Shengwei Lai, Chen Li, Wen Tian

**Affiliations:** Department of Thyroid & Hernia Surgery, Medical Department of General Surgery, Chinese People’s Liberation Army General Hospital, Beijing, China

**Keywords:** poorly differentiated thyroid carcinoma, recurrence-free survival, overall survival, cancer specific survival, decision curve analysis, X-tile

## Abstract

**Objective:**

To elucidate the clinicopathological characteristics and prognostic factors of poorly differentiated thyroid carcinoma.

**Method:**

A total of 24912 thyroid carcinoma patients admitted to the First Medical Center of Chinese People’s Liberation Army General Hospital from 2005 to 2020 were retrospectively reviewed. A total of 94 patients (39 males and 55 females, a male-female ratio of 1:1.4) fulfilled the selection criteria. Of these, 73 patients had undergone surgery. The clinical and pathological data were collected from each enrolled patient. Univariate and multivariate Cox regression analyses were performed to determine independent prognostic factors. All analyses were performed with the SPSS version 26.0 and R version 1.2.5033 in the R Studio environment.

**Results:**

The specimens included 20 cases of poorly differentiated thyroid carcinoma complicated with papillary thyroid carcinoma, 17 cases complicated with follicular thyroid carcinoma, 34 cases complicated with other pathological types and 23 with a separate entity. The patient demonstrated a large age span, median age was 57 years (range 8–85 years, average 55.20 ± 15.74 years). The survival time of the 94 cases was calculated, and the mean Overall survival time was 33 (range, 1-170) months, and the mean Recurrence-free survival time was 14 (range, 1-90) months. Recurrence-free mortality is related to the age at diagnosis, extrathyroidal extension and Associated thyroid cancer (p<0.05). In contrast, overall mortality is related to the age at diagnosis, sex, extrathyroidal extension, T stage (AJCC 8th), surgery and radiation (p<0.05).

**Conclusion:**

Middle-aged and elderly patients are still at high risk for poorly differentiated thyroid carcinoma. The pathologic results of poorly differentiated thyroid carcinoma are varied, and reasonable treatment has an important impact on the prognosis of poorly differentiated thyroid carcinoma.

## Introduction

Poorly differentiated thyroid carcinoma accounts for 0.2-7% of all malignant thyroid tumors and independent thyroid cancer histocyte, first introduced by Japanese scholar Sakamoto et al. in 1983 (1–3). The characteristic histology was the presence of solid, trabecular and/or scirrhous patterns. Carcangiu et al. (4) described 25 cases of poorly differentiated thyroid carcinoma, first defined as insular carcinoma and histopathologic diagnostic criteria in 1984. After that, we found accurate clinicopathological characteristics and suitable treatment options are a hot topic of scholars worldwide. It was classified by the World Health Organization in 2004 as (5) “a tumor of follicular cell origin with morphologic and biologic attributes intermediate between differentiated thyroid carcinoma and anaplastic thyroid carcinoma.” In 2006, the international conference was held in Turin, Italy, and issued the poorly differentiated thyroid carcinoma diagnostic criteria (Turin- poorly differentiated thyroid carcinoma) (6). The diagnostic criteria of poorly differentiated thyroid carcinoma based on the Turin consensus must meet the following diagnostic criteria: i) a solid/trabecular/insular pattern of growth, ii) absence of conventional nuclear features of papillary carcinoma, and iii) presence of at least one of the following features: convoluted nuclei, mitotic activity > 3/10 high power field, or necrosis. However, given the newness of this carcinoma, most studies on poorly differentiated thyroid carcinoma were presented in the format of a literature review or case report, which focused on the genetic testing of the carcinoma. Based on our prior knowledge of poorly differentiated thyroid carcinoma, this study intended to report the experience of the First Medical Center of Chinese People’s Liberation Army General Hospital in China by reporting the poorly differentiated thyroid carcinoma’ s predictive factors and clinicopathological characteristics.

## Materials and methods

### Study design and patients

**Figure 1:**
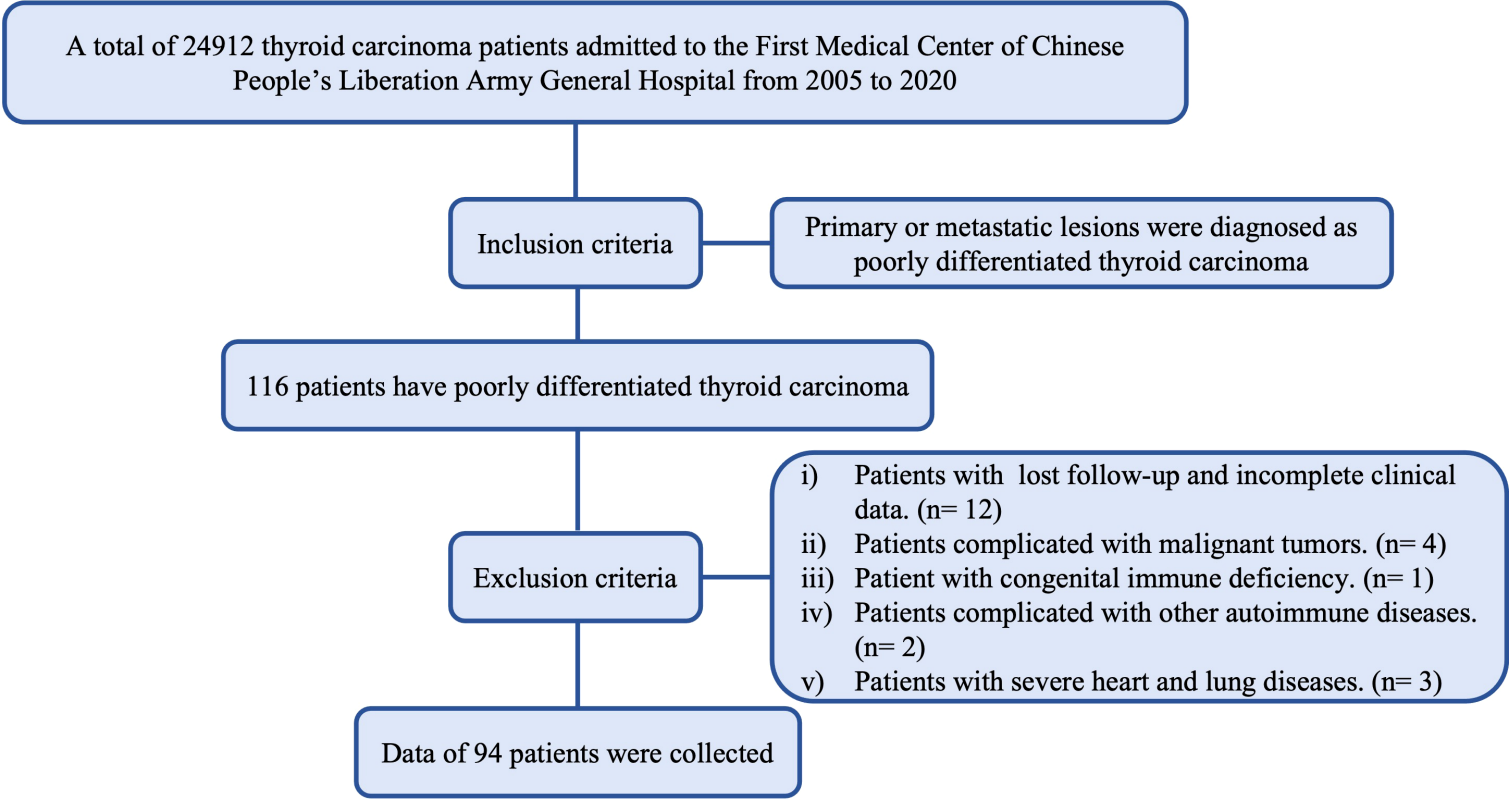
Flowchart of the inclusion and exclusion of patients.

A total of 24912 thyroid carcinoma patients admitted to the First Medical Center of Chinese People’s Liberation Army General Hospital from 2005 to 2020 were retrospectively reviewed. 94 patients (39 males and 55 females, with a male-female ratio of 1:1.4) fulfilled the selection criteria (**Figure 1**). The Institutional Review Board approved the study. The considered variables included year of diagnosis, age at diagnosis, sex, extrathyroidal extension, multifocality, AJCC 8th staging (7) information, surgical approach, radiation, Chemotherapy, associated thyroid cancer, survival months and vital status. The inclusion criteria were histological diagnosis of a primary solid lesion or metastatic lesions. The exclusion criteria were as follows: i) Patients with lost follow-up and incomplete clinical data; ii) Patients complicated with malignant tumors. iii) Patient with congenital immune deficiency; iv) Patients complicated with other autoimmune diserses; v) Patients with severe heart and lung diseases.

### Management and follow-up protocols

After the surgery or Biopsy, the patients were followed up at the outpatient department for one month, three months, six months during the first year, and then six months after using the same modalities. Recurrence-free survival was measured from the date of surgery to the date of first recurrence or last follow-up, whereas Overall survival was defined as the interval between the date of the first diagnosis of poorly differentiated thyroid carcinoma and the date of death or last follow-up. Among these, 94 cases were used. We followed the Turin diagnostic criteria for the diagnosis of poorly differentiated thyroid carcinoma. The cytomorphologic features were compared with histopathologic features. Experienced histopathologists judged all pathological results in our hospital’s pathology department.

### Statistical analysis

Inferential statistics were used to analyze the essential characteristics of the selected patients with poorly differentiated thyroid carcinoma. We compared categorical variables using Pearson χ2 or the exact Fisher’s tests between groups, whereas analyses of variance were used for continuous variables. The optimal cut-off values for continuous variables (e.g., year of diagnosis, age at diagnosis) were determined using the X-tile program (Yale University, New Haven, Connecticut, USA). Univariate and multivariate regression analyses were performed utilizing the Cox proportional hazards regression model to analyze the prognosis’s independent factors. Meanwhile, statistical significance was reached at a 95% confidence interval (p < 0.05). To estimate survival probability, Recurrence-free survival and Overall survival were assessed using Multivariable Cox regression and the Kaplan-Meier curve. All analyses were performed with the SPSS version 26.0 (IBM Corp., Armonk, NY) and R version 1.2.5033 (The R Development Core Team, Vienna, Austria) in the R Studio environment.

## Results

### Demographic and clinicopathological characteristics of poorly differentiated thyroid carcinoma

The patient demonstrated a large age span. The median age was 57 (range 8–85 years, average 55.20 ± 15.74 years). Tumor stage (TNM stage, AJCC 8th) were mostly T4 (n=54, 57.4%), N1 (n=78, 83.0%) and M0 (n=71, 75.5%). 73 patients had surgical treatment, 44 (46.8%) underwent total thyroidectomy, and 29 (30.9%) underwent thyroidectomy + lymph node dissection. 49 (77.7%) patients received radiotherapy, 23 (24.5%) Radioactive iodine and 26 (27.7%) External beam radiotherapy. 22 (23.4%) patients received chemotherapy. 7 patients were subjected to all three treatments (surgery, radiotherapy and chemotherapy); The survival time of the 94 cases was calculated, the mean Overall survival time was 33 (range, 1-170) months, and the mean Recurrence-free survival time was 14 (range, 1-90) months (**Table 1**).

**Table 1:** Demographic and clinicopathological characteristics of poorly differentiated thyroid carcinoma.

Patient number	n	Patient number	n
Median age (y) (min-max)	57(8-85)	M0	71(75.5%)
Median year (y) (min-max)	2017(2005-2020)	M1	23(24.5%)
Sex, n (%)		Surgery, n (%)	
Male	39(41.5%)	Biopsy or no surgery	21(22.3%)
Female	55(58.5%)	Thyroidectomy only	44(46.8%)
Extrathyroidal extension, n(%)		Thyroidectomy +lymph node dissection	29(30.9%)
None	11(11.7%)	Radiation, n (%)	
Yes	83(88.3%)	None or refused	45(47.9%)
Multifocality,n (%)		Radioactive iodine	23(24.5%)
None	60(63.8%)	External beam radiotherapy	26(27.7%)
Yes	34(36.2%)	Chemotherapy, n (%)	
T stage (AJCC 8th), n (%)		None or unknown	72(76.6%)
T1	18(19.1%)	Yes	22(23.4%)
T2	15(16.0%)	Associated thyroid cancer	
T3	7(7.4%)	Papillary only	20(21.3%)
T4	54(57.4%)	Follicular only	17(18.9%)
N stage (AJCC 8th), n (%)		Other	34(36.2%)
N0	16(17.0%)	None	23(24.5%)
N1	78(83.0%)	Median recurrence-free survival time (m) (min-max)	14(1-90)
M stage (AJCC 8th), n (%)		Median overall survival time (m) (min-max)	33(1-170)

### Risk factors for survival

**Figure 2:**
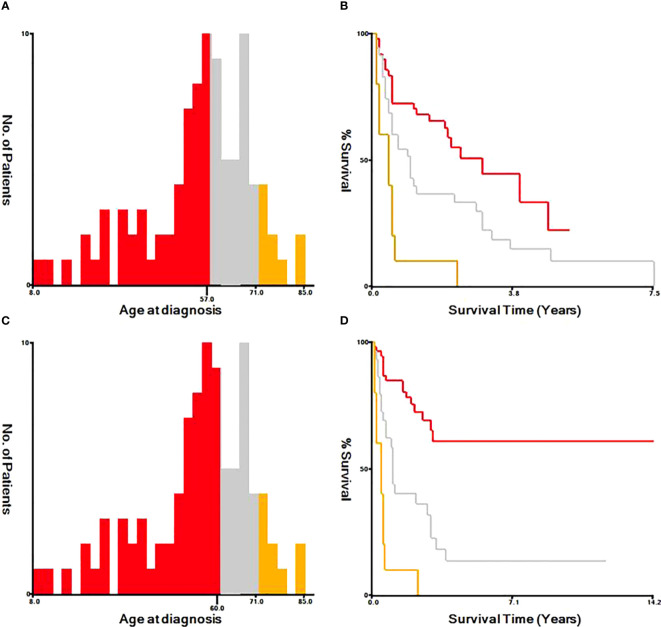
Identification of optimal cut-off values of age at diagnosis by X-tile program. The optimal cut-off value for the age at diagnosis were identified as 57 years and 71 years based on recurrence-free survival **(A, B)**. The optimal cut-off value for the age at diagnosis were identified as 60 years and 71 years based on overall survival **(C, D)**.

**Figure 3:**
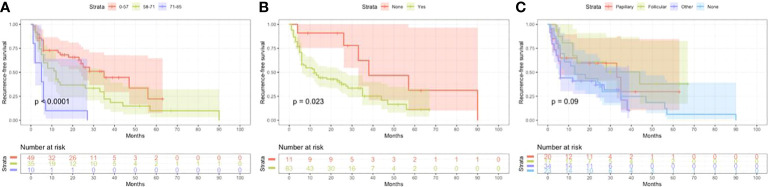
Kaplan Meier curves of recurrence-free survival mortality for matched poorly differentiated thyroid carcinoma. Age at diagnosis **(A)**, Extrathyroidal extension **(B)**, Associated thyroid carcinoma **(C)**.

**Figure 4:**
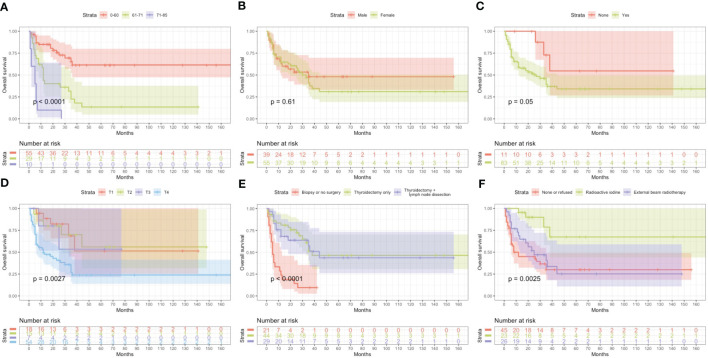
Kaplan Meier curves of overall survival mortality for matched poorly differentiated thyroid carcinoma. Age at diagosis **(A)**, Sex **(B)**, Extrathyroidal extension **(C)**, T stage AJCC 8th **(D)**, Surgery **(E)**, Radiation **(F)**.

Based on univariate and multivariate Cox regression analysis in **Table 2** or **3**, independent prognostic factors of recurrence-free mortality and overall mortality of poorly differentiated thyroid carcinoma were determined, respectively. The significant variables in the univariate analysis were entered into multivariate methods. Recurrence-free mortality is related to the age at diagnosis (p=0.014), extrathyroidal extension (p=0.034) and Associated thyroid cancer (p=0.052), while overall mortality is related to the age at diagnosis (p=<0.001), sex (p=0.049), extrathyroidal extension (p=0.006), T stage (AJCC 8th) (p=0.014), surgery (p=0.023) and radiation (p=0.029). The independent continuous variable (age at diagnosis) is a categorical variable by the x-tile program (**Figure 2**). The survival curves were plotted by the Kaplan-Meier curve (**Figures 3**, **4**) method to compare independent prognostic factors of poorly differentiated thyroid carcinoma.

**Table 2:** Univariate cox regression analyses: outcome of poorly differentiated thyroid carcinoma recurrence-free survival and overall survival.

Variable	Recurrence-free survival		Overall survival	
	Hazard Ratio (95% CI)	p-value	Hazard Ratio (95% CI)	p-value
Age at diagnosis (year)	1.039(1.017-1.061)	<0.001	1.031(1.006-1.057)	0.014
Year of diagnosis (year)	0.962(0.894-1.036)	0.305	0.934(0.850-1.026)	0.153
Sex				
Male	1		1	
Female	0.875(0.525-1.458)	0.608	1.188(0.634-2.227)	0.591
Extrathyroidal extension				
None	1		1	
Yes	2.758(1.087-6.997)	0.033	3.662(1.100-12.187)	0.034
Multifocality				
None	1		1	
Yes	0.895(0.528-1.518)	0.681	1.235(0.657-2.319)	0.512
T stage (AJCC 8th)				
T1	1		1	
T2	1.599(0.577-4.427)	0.367	0.955(0.272-3.353)	0.943
T3	0.993(0.254-3.885)	0.992	0.933(0.219-3.974)	0.925
T4	3.601(1.583-8.191)	0.002	1.181(0.395-3.526)	0.766
N stage (AJCC 8th)				
N0	1		1	
N1	3.751(1.358-10.358)	0.011	2.230(0.657-7.566)	0.198
M stage (AJCC 8th)				
M0	1		1	
M1	2.197(1.277-3.780)	0.004	1.739(0.834-3.623)	0.140
Surgery				
Biopsy or no surgery	1		1	
Thyroidectomy only	0.302(0.161-0.567)	<0.001	0.579(0.275-1.215)	0.148
Thyroidectomy + lymph node dissection	0.493(0.262-0.928)	0.028	0.692(0.309-1.550)	0.371
Radiation				
None or refused	1		1	
Radioactive iodine	0.232(0.103-0.523)	<0.001	0.487(0.186-1.273)	0.142
External beam radiotherapy	0.706(0.404-1.235)	0.223	0.845(0.437-1.633)	0.617
Chemotherapy				
None or unknown	1		1	
Yes	1.255(0.694-2.269)	0.452	0.670(0.333-1.345)	0.260
Associated thyroid cancer				
Papillary only	1		1	
Follicular only	0.767(0.301-1.954)	0.579	0.317(0.099-1.012)	0.052
Other	1.860(0.886-3.903)	0.101	1.076(0.460-2.516)	0.865
None	1.500(0.699-3.219)	0.298	0.820(0.624-2.076)	0.676

**Table 3:** Multivariate cox regression analyses: outcome of poorly differentiated thyroid carcinoma recurrence-free survival and overall survival.

Variable	Recurrence-free survival		Overall survival	
	Hazard Ratio (95% CI)	p-value	Hazard Ratio (95% CI)	p-value
Age at diagnosis (year)	1.084(1.053-1.117)	<0.001	1.075(1.036-1.114)	<0.001
Year of diagnosis (year)	1.034(0.942-1.135)	0.477	0.987(0.869-1.120)	0.835
Sex				
Male	1		1	
Female	1.160(0.649-2.075)	0.616	2.110(1.003-4.439)	0.049
Extrathyroidal extension				
None	1		1	
Yes	2.986(0.927-9.621)	0.067	8.088(1.825-35.844)	0.006
Multifocality				
None	1		1	
Yes	0,934(0.519-1.683)	0,820	1.724(0.864-3.439)	0.122
T stage (AJCC 8th)				
T1	1		1	
T2	1.100(0.318-3.807)	0.880	0.120(0.022-0.648)	0.014
T3	1.226(0.238-6.329)	0.808	0.504(0.074-3.430)	0.484
T4	3.446(1.352-8.784)	0.010	0.335(0.085-1.314)	0.117
N stage (AJCC 8th)				
N0	1		1	
N1	3.158(0.980-10.177)	0.054	1.492(0.354-6.291)	0.585
M stage (AJCC 8th)				
M0	1		1	
M1	2.646(1.486-4.7130	0.001	1.647(0.730-3.718)	0.229
Surgery				
Biopsy or no surgery	1		1	
Thyroidectomy only	0.207(0.106-0.405)	<0.001	0.416(0.173-0.999)	0.050
Thyroidectomy + lymph node dissection	0.242(0.118-0.498)	<0.001	0.333(0.129-0.860)	0.023
Radiation				
None or refused	1		1	
Radioactive iodine	0.192(0.067-0.547)	0.002	0.237(0.065-0.864)	0.029
External beam radiotherapy	0.780(0.423-1.438)	0.426	1.104(0.505-2.414)	0.804
Chemotherapy				
None or unknown	1		1	
Yes	1.069(0.556-2.055)	0.842	0.580(0.259-1.301)	0.186
Associated thyroid cancer				
Papillary only	1		1	
Follicular only	1.228(0.430-3.502)	0.701	0.400(0.110-1.450)	0.163
Other	2.496(1.059-5.883)	0.037	1.329(0.486-3.630)	0.579
None	1.750(0.705-4.343)	0.228	1.079(0.341-3.415)	0.897

### Histopathological characteristics of poorly differentiated thyroid carcinoma

**Figure 5:**
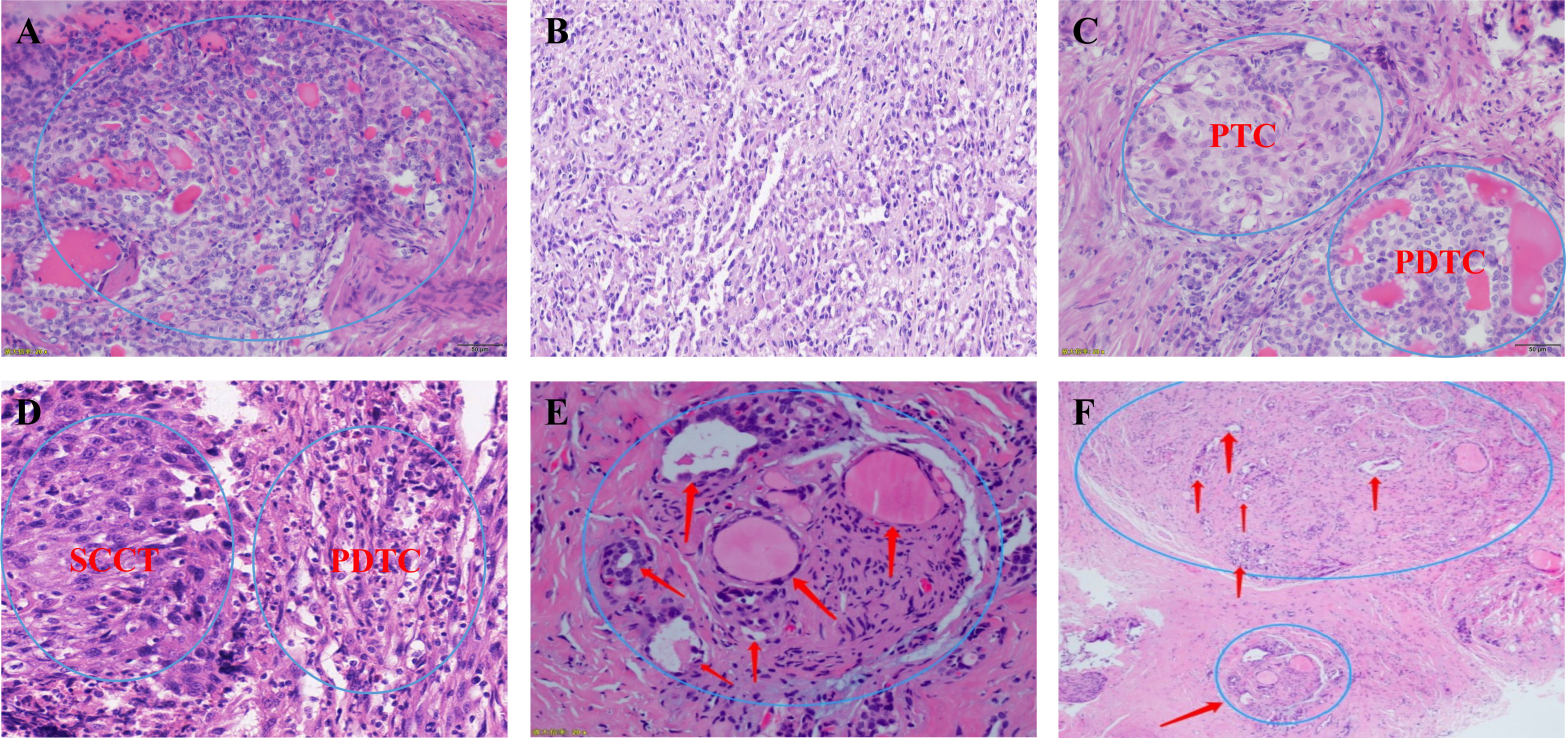
**(A, B)** Microscopic appearance of small cell type poorly differentiated thyroid carcinoma; **(C)** Microscopic appearance of small cell type mixed subtype thyroid carcinoma papillary thyroid carcinoma + poorly differentiated thyroid carcinoma; **(D)** Microscopic appearance of small cell type mixed subtype thyroid carcinoma squamous cell carcinoma of the thyroid + poorly differentiated thyroid carcinoma; **(E, F)** Schematic drawing shows invasion of the recurrent laryngeal nerve by poorly differentiated thyroid carcinoma. PTC, papillary thyroid carcinoma; PDTC, poorly differentiated thyroid carcinoma; SCCT, squamous cell carcinoma of the thyroid;.

All patients were diagnosed by postoperative pathology. We first looked at the gross pathology of the poorly differentiated thyroid carcinoma. The carcinomas are large (median size: 4.5 cm), solid, and light brown to grey. Some show soft, pale areas of necrosis. Extension beyond the thyroid capsule is standard, and resection margins are often positive, but the extrathyroidal extension is less pervasive than anaplastic thyroid carcinoma. The specimens included 20 (21.3%) cases of poorly differentiated thyroid carcinoma complicated with papillary thyroid carcinoma, 17 (18.9%) cases complicated with follicular thyroid carcinoma, 34 (36.2%) cases complicated with other pathological types (e.g., mixed subtype thyroid carcinoma) and 23 (24.5%) with a separate entity (**Figure 5**).

## Discussion

Our study report is the largest retrospective population-based poorly differentiated thyroid carcinoma screening cohort published in Asia with homogeneous and consensual diagnostic criteria. It confirms that poorly differentiated thyroid carcinoma presents more wildly invasive and local recurrence rates than classical differentiated thyroid carcinoma (8–10). During the median follow-up of 25.5 months (range, 1–170), the median Recurrence-free survival time and median Overall survival time are 14 (range, 1-90) and 33 (range, 1-170), respectively. In our study, In our study, the incidence of poorly differentiated thyroid carcinoma was 0.38%. Iodine deficiency may be a contributing environmental and/or genetic factor, given the association of poorly differentiated thyroid carcinoma with nodular goiter. In parts of America, the incidence of poorly differentiated thyroid carcinoma is up to 15% (11), and there is no association with radiation exposure. Alternatively, some have argued that the poorly differentiated thyroid carcinoma arises because of a loss of differentiation (synchronous or metachronous) of differentiated thyroid carcinoma (often of the follicular variant) (12), and others appear *de novo*.

In the Cox proportional hazards regression model, we found statistically significant differences when comparing age at diagnosis and sex. The risk of mortality in males is more than twice that of females. The optimal cut-off value for the age at diagnosis was identified as 57, 71 years old, and 60, 71 years old based on Recurrence-free survival and Overall survival by X-tile program, respectively. Kazaure (13) reported that patients with poorly differentiated thyroid carcinoma were more elderly than those with differentiated thyroid carcinoma, similar to our study. Thus, middle-aged and elderly male patients are at high risk of poorly differentiated thyroid carcinoma. It is considered that the mid-late stage already causes most male patients with tumors, and thyroid cancer in elderly age may have high-grade biologic aggressiveness. Furthermore, univariate and multivariate analyses found that locally invasive extrathyroidal is an independent risk factor for Recurrence-free survival and Overall survival. Unlike differentiated thyroid carcinoma, 83% of poorly differentiated thyroid carcinoma patients tend to present with the locally invasive extrathyroidal disease.

To date, the treatment of poorly differentiated thyroid carcinoma has not been standardized due to the rarity and heterogeneous nature of this disease. Therefore, treatment is mainly extrapolated from the treatment experience of differentiated thyroid carcinoma, and surgery is the first choice. However, poorly differentiated thyroid carcinoma has late clinical stages when discovered, and they often have cervical lymph node metastasis and distant metastasis. The tumors overgrow and are not separated from the surrounding vital tissues (trachea, esophagus, nerves, arteries and veins, etc.).The surgical method is more complex, and patients often lose the opportunity for surgery. Univariate and multivariate analysis found that the surgical method considerably influences differentiation (p<0.05). Total thyroidectomy plus lymph node dissection can reduce recurrence and mortality. Therefore, it is necessary to assess whether radical surgery can be performed before surgery accurately, which is an essential prerequisite for low recurrence and mortality of poorly differentiated thyroid carcinoma.

According to the World Health Organization studies, poorly differentiated thyroid carcinoma prognosis is generally inferior, with a 5-year survival rate of only 50%, and most patients die within 2 years after surgery (14). The clinical benefit of radioactive iodine, external beam radiotherapy and chemotherapy in survival is yet to be proved, and the efficacy has been questioned. In this study, it was found that radioactive iodine can effectively offers the potential to decrease mortality in poorly differentiated thyroid carcinoma (p<0.05). However, only 23 people (24.5%) received radioactive iodine after surgery, possibly because the clinical diagnosis did not correspond with pathological findings in some cases (15). This study found that the response rate of external beam radiotherapy or chemotherapy alone is low, and the prognosis is not significantly improved (p>0.05). poorly differentiated thyroid carcinoma cases more frequently displayed distant metastasis with little evidence of primary lesions or regional lymph node metastasis that cannot be removed due to surgery. No evidence shows that poorly differentiated thyroid carcinoma has a higher local recurrence rate than differentiated thyroid carcinoma. Therefore, it should be individualized for the individual patients (16). In the case of poorly differentiated thyroid carcinoma with large tumors, especially those with external invasion or internal jugular vein tumor thrombosis, external beam radiotherapy or chemotherapy could be given. It is not advisable to take significant risks during surgical treatment (such as resection of the carotid artery) or multiple organ resections (such as larynx, hypopharynx, esophagus, etc.) for a radical cure. Therefore, poorly differentiated thyroid carcinoma undergoes radical surgical resection to improve the local control rate and survival rate, and at the same time, individualized treatment standards are supplemented after an operation.

Our study found that 75.5% of poorly differentiated thyroid carcinoma patients had mixed subtype thyroid carcinoma. This rare type of thyroid carcinoma shows two or more types of mixed pathology within the same tumor. In addition, we found that thyroid cancer tissue with cytological characteristics of other classes was mixed in poorly differentiated thyroid carcinoma. Therefore, poorly differentiated thyroid carcinoma is often associated with different pathologic types. This study’s cox multivariate analysis indicated that the concomitant pathological type is an independent risk factor for Recurrence-free survival (p<0.05). Meanwhile, when poorly differentiated thyroid carcinoma is mixed with other types of thyroid cancer (such as anaplastic thyroid carcinoma) and it has the highest degree of malignancy, the continuous dedifferentiation of thyroid cancer was considered the cause (17).

In summary, poorly differentiated thyroid carcinoma is a highly aggressive malignant tumor. It has paid enough attention to poorly differentiated thyroid carcinoma. Meanwhile, with the further exploration of the molecular mechanism of poorly differentiated thyroid carcinomaand the development of clinical technology, the means of tumor treatment are improved and perfected constantly. Treatment should be individualized and generally multidisciplinary to improve the prognosis of patients with poorly differentiated thyroid carcinoma. In the next few years, we are confident that poorly differentiated thyroid carcinoma will have more accurate therapeutic options and achieve a superior clinical outcome.

## Data availability statement

The raw data supporting the conclusions of this article will be made available by the authors, without undue reservation.

## Ethics statement

The studies involving human participants were reviewed and approved by Ethics Committee of Chinese PLA General Hospital. The patients/participants provided their written informed consent to participate in this study.

## Author contributions

ZW, BW conceived, designed and wrote the initial draft of the manuscript. ZW, QL, JY, BW, CL, SH and SL performed the statistical analyses. WT, ZW and BW reviewed, revised and approved the final version of the manuscript. All authors contributed to the article and approved the submitted version.
